# Case Report: Multimodal management of a rare pediatric astroblastoma using proton beam therapy and Gamma Knife radiosurgery—a case report and literature review

**DOI:** 10.3389/fonc.2025.1620637

**Published:** 2025-09-04

**Authors:** Xijia Zhang, Xinfa Wang, Weihai Ning, Jianfeng Liang, Yonglong Jin, Yuqian Nie, Jie Wang, Zishen Wang, Wei Wang, Jun Zhao, Jingjing Kong, Lu Yang, Dongxue Zhou, Yue Zou, Masashi Mizumoto, Shosei Shimizu

**Affiliations:** ^1^ Department of Neurosurgery, Beijing Tiantan Hospital of Capital Medical University, Beijing, China; ^2^ Department of Neurosurgery, Children’s Hospital of Nanjing Medical University, Nanjing, Jiangsu, China; ^3^ Department of Neurosurgery, Sanbo Brain Hospital, Capital Medical University, Beijing, China; ^4^ Department of Neurosurgery, Peking University International Hospital, Beijing, China; ^5^ Department of Radiotherapy, The Affiliated Hospital of Qingdao University, Qingdao, Shandong, China; ^6^ Department of Pediatric Radiation Therapy Center, Hebei Yizhou Cancer Hospital, Zhuozhou, Hebei, China; ^7^ Department of Radiotherapy Physics and Technology, Hebei Yizhou Cancer Hospital, Zhuozhou, Hebei, China; ^8^ Department of Radiology, Hebei Yizhou Cancer Hospital, Zhuozhou, Hebei, China; ^9^ Department of Radiation Oncology, University of Tsukuba, Tsukuba, Japan

**Keywords:** astroblastoma, proton beam therapy, Gamma Knife radiosurgery, pediatric oncology, case report

## Abstract

**Introduction:**

Astroblastoma is an infrequent glial tumor, with the MN1-altered subtype recognized in the 2021 WHO classification. This report details the management of a 4-year-old girl diagnosed with CNS WHO Grade 3 MN1-altered astroblastoma, also found to have a heterozygous BRCA2 mutation. We highlight a sequential multimodal treatment approach involving proton beam therapy (PBT), targeted chemotherapy with a PARP inhibitor, and subsequent salvage Gamma Knife radiosurgery (GKRS).

**Main symptoms and findings:**

The patient presented with right lower extremity weakness and gait disturbance. Initial treatment involved maximal safe resection followed by adjuvant PBT (craniospinal irradiation 36 Gy, local boost to 54 Gy). PBT was selected for its dosimetric advantages, notably minimizing radiation dose to surrounding healthy tissues, thereby reducing potential acute toxicity and long-term risks compared to conventional photon therapy. Despite this, residual tumor persisted. Following the discovery of a BRCA2 mutation, the PARP inhibitor fluzoparib was administered, which was associated with temporary disease stabilization.

**Diagnoses, interventions, outcomes:**

After a second resection confirming residual disease, salvage stereotactic radiosurgery (SRS) using Gamma Knife (30 Gy in 5 fractions) was administered to the remaining lesions. The patient has demonstrated sustained local control with no tumor progression for over 18 months post-SRS, with only mild, asymptomatic perilesional edema and no neurological deficits.

**Conclusion - Take-away lesson:**

This case suggests that leveraging the tissue-sparing benefits of initial PBT may enable effective salvage SRS for managing residual or recurrent high-grade pediatric astroblastoma. Furthermore, it highlights the potential role of molecular profiling to guide targeted therapies in these rare tumors.

## Introduction

Astroblastoma represents a rare and infrequent subset of glial tumors within the central nervous system, with an estimated incidence of 0.45% to 2.8% among all neurological tumors (1, 2). While historically considered a predominantly pediatric brain tumor, comprehensive analyses have revealed a bimodal age distribution, exhibiting incidence peaks in both childhood (5–10 years) and young adulthood (21–30 years) (1, 3, 4). Furthermore, a notable female predilection has been consistently observed, with reported male-to-female ratios approximating 1:11 (3, 4). Traditionally, astroblastoma has lacked a specific World Health Organization (WHO) grade due to its intrinsic variability in biological behavior (2, 5). Histopathologically, these tumors have been broadly categorized into “low-grade” or “well-differentiated” variants, generally associated with a more favorable prognosis, and “high-grade” or “anaplastic” counterparts, which typically portend poorer clinical outcomes (6, 7). The clinical spectrum of astroblastoma is diverse, encompassing tumors with relatively indolent progression to those exhibiting aggressive malignant characteristics (2, 3, 8, 9). Notably, the 2021 WHO classification of central nervous system tumors has integrated molecular diagnostics, recognizing “astroblastoma, MN1-altered” as a distinct entity within the category of circumscribed astrocytic gliomas, characterized by the presence of meningioma 1 (MN1) gene alterations (7, 10).

In the management of astroblastomas, the current body of evidence, largely derived from case reports and retrospective analyses, underscores the critical role of maximal safe surgical resection as the primary intervention, aiming to achieve gross total resection (GTR) which correlates with improved tumor control rates and progression-free survival (8, 9, 11–15). Subtotal resection (STR) is generally discouraged as it may not provide equivalent tumor control and often necessitates the consideration of adjuvant therapies (14, 15). The utility of adjuvant radiation therapy (RT), particularly in the context of high-grade and recurrent astroblastomas, suggests a potential survival benefit with postoperative RT (2, 8, 9, 11, 13, 16, 17). However, the optimal dose and target volumes remain under investigation. The role of adjuvant chemotherapy in the management of astroblastoma is even less well-defined, with conflicting reports and a general consensus of unclear benefit in both low- and high-grade tumors, although temozolomide-based regimens have shown some promise in isolated cases (2, 5, 8, 18). This report presents a unique case of high-grade pediatric astroblastoma managed with sequential PBT and GKRS, highlighting the potential feasibility and benefit of this multimodal approach, particularly the role of initial PBT in enabling safe re-irradiation for residual disease and the use of targeted therapy guided by molecular profiling.

## Clinical summary

**Figure 1:**
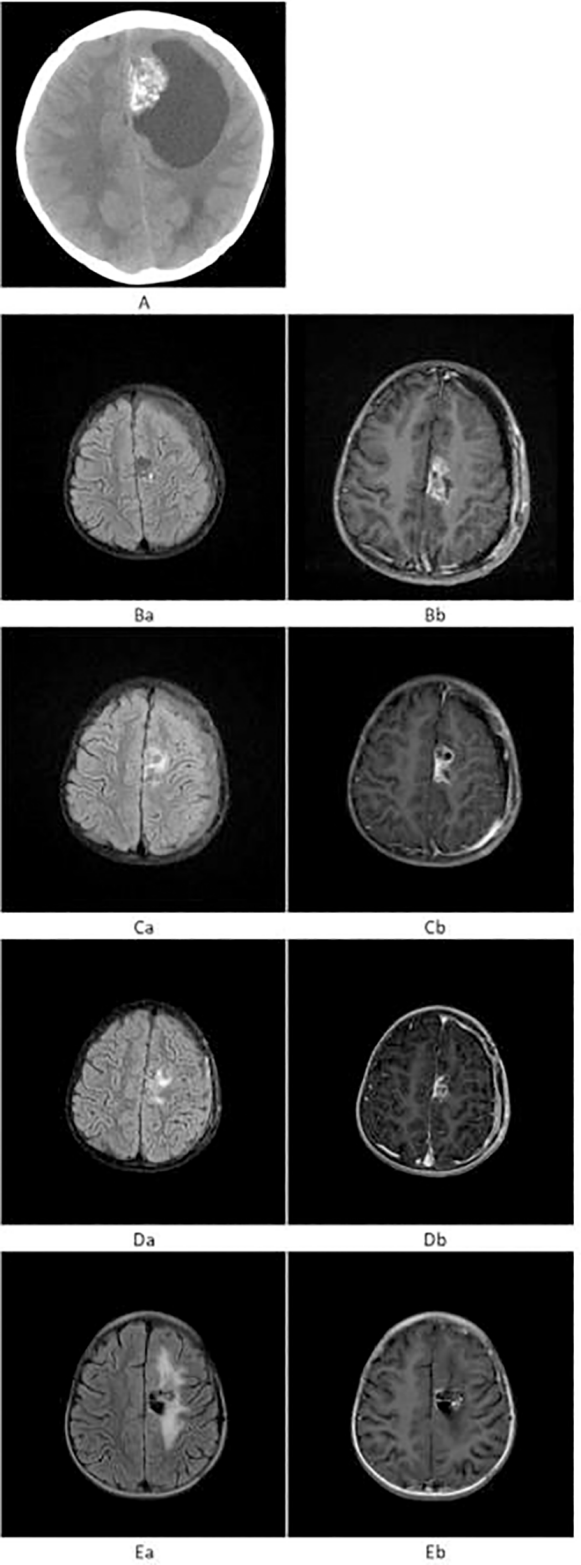
**(A)** Preoperative axial non-contrast CT showing a patchy, heterogeneous hyperdense lesion in the left frontal lobe with surrounding cystic hypodensity. **(B)** Prior to proton beam therapy. **(Ba)** T2-FLAIR shows patchy mild hyperintensity in the left frontal lobe. **(Bb)** T1WI+C reveals heterogeneous mild-to-moderate enhancement of the lesion. **(C)** After proton beam therapy. **(Ca)** T2-FLAIR shows persistent mild hyperintensity. **(Cb)** T1WI+C demonstrates reduced enhancement of the lesion, with new perilesional brain edema. **(D)** At 9 months post-proton beam therapy. **(Da)** T2-FLAIR shows mild patchy hyperintensity. **(Db)** T1WI+C shows reduced size and persistent mild enhancement. **(E)** At 18 months post-Gamma Knife radiosurgery. **(Ea)** T2-FLAIR shows surrounding mild hyperintensity. **(Eb)** T1WI+C reveals no solid enhancement, with only linear enhancement along the cystic margin.

This case involves a 4-year-old girl diagnosed with astroblastoma harboring MN1 alteration, classified as a CNS WHO Grade 3 tumor. At initial presentation, she experienced right lower extremity weakness and gait disturbance. The patient had no significant past medical history, no history of psychiatric illness, and had met age-appropriate developmental milestones prior to presentation. Family history was non-contributory for neurological disorders or malignancy. Further psychosocial details are withheld for privacy. The patient had no relevant past interventions prior to the diagnosis of astroblastoma. **Table 1** summarizes the key events during the patient’s episode of care. The sequence of imaging findings corresponding to key time points is shown in **Figure 1**.

**Table 1:** Timeline summarizing key clinical events.

Timeline summarizing key clinical events		
Time Point (Approximate)	Key Event	Corresponding Figure
May 2022 (Month 0)	Onset of symptoms (right leg weakness, unsteady gait); Worsening despite conservative treatment.	
Late May 2022 (Month 0)	Initial CT scan reveals lesion; Craniotomy & maximal safe resection (Surgery 1); Post-op MRI shows residual.	**Figure 1A** (Pre-op CT)
June 2022 (Month 0-1)	Histopathological Diagnosis: MN1-altered Astroblastoma, CNS WHO Grade 3.	
July-Aug 2022 (Month 1-3)	Adjuvant Proton Beam Therapy (PBT): CSI (36 Gy/20fx) + Local Boost (18 Gy/10fx, Total 54 Gy).	**Figure 1B** (Pre-PBT MRI). **Figure 2** (PBT Plan)
Aug 2022 (Month 3)	Post-PBT MRI: Reduced enhancement, new perilesional edema.	**Figure 1C**
Sep 2022 (Month 4)	Initiation of oral chemotherapy (Fluzoparib) due to ambiguous MRI findings; Temporary stabilization.	
May 2023 (Month 12)	Follow-up MRI (~9 months post-PBT): Decreased solid component size, persistent mild enhancement.	**Figure 1D**
June 2023 (Month 13)	Second Craniotomy & Resection; Histopathology confirms residual astroblastoma.	
July 2023 (Month 14)	Salvage Gamma Knife Radiosurgery (GKRS): 30 Gy/5fx.	**Figure 3** (GKRS Plan)
Jan 2025 (Month 32)	Last Follow-up (~18 months post-GKRS): MRI shows stable post-treatment changes; No recurrence; No deficits.	**Figure 1E**

In May 2022, the patient developed right lower extremity weakness and unsteady gait without obvious precipitating factors. She was initially diagnosed with synovitis at a local hospital and received conservative treatment, including rest and physical therapy, without improvement. But the weakness gradually worsened. Initial neurological examination confirmed these reported symptoms, although specific details like strength grading were not available. Pre-treatment non-contrast axial CT, performed due to worsening symptoms, revealed a patchy, heterogeneous hyperdense lesion in the left frontal lobe, surrounded by a cystic hypodense area (**Figure 1A**). Craniotomy and tumor resection were performed, and postoperative MRI revealed a small residual enhancing lesion. Histopathological analysis confirmed the diagnosis of MN1-altered astroblastoma (CNS WHO Grade 3).

**Figure 2:**
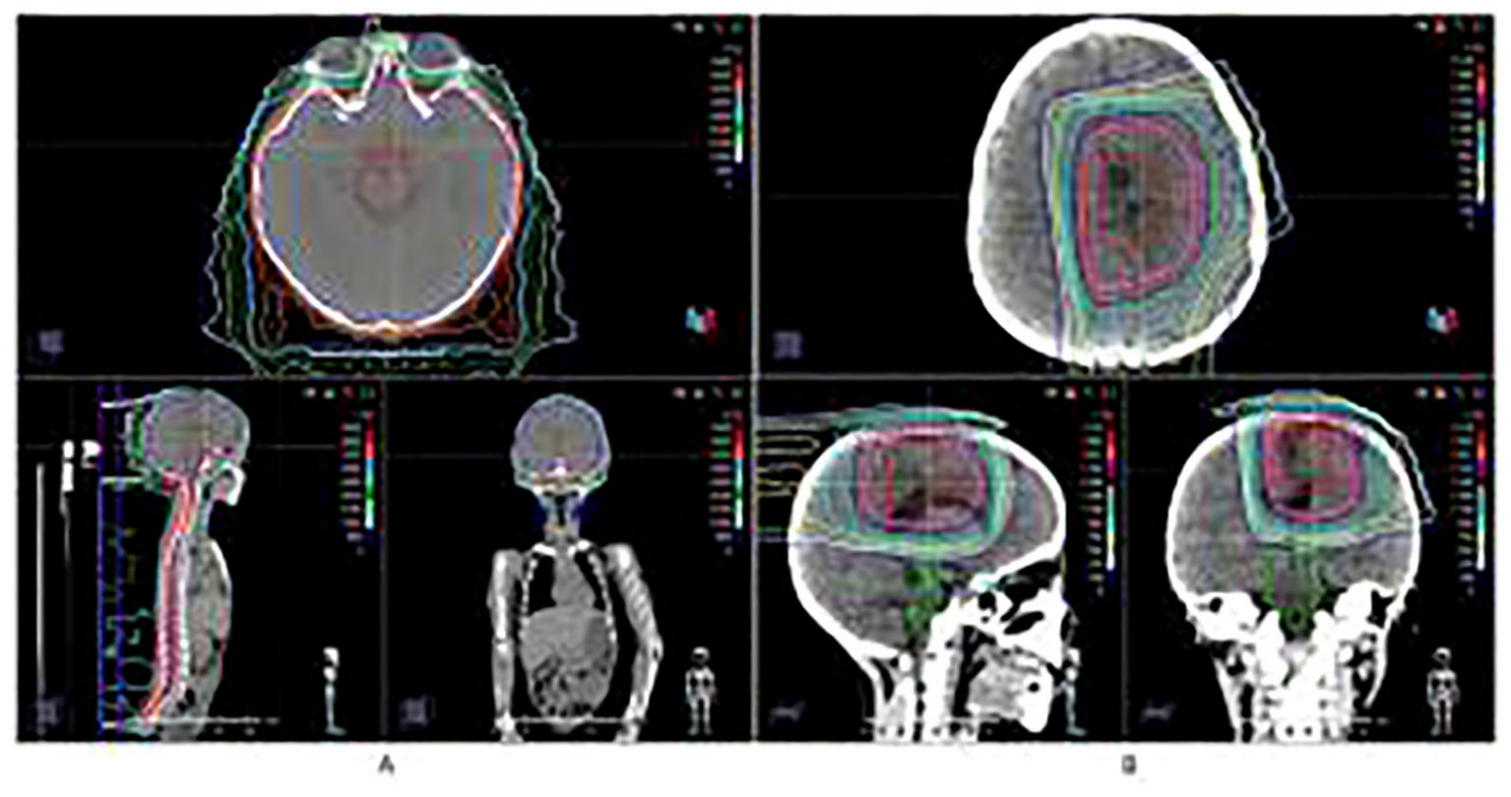
Proton Beam Therapy treatment plan and dose distribution. **(A)** Craniospinal irradiation (CSI) at 36 Gy in 20 fractions. **(B)** Local boost of 18 Gy in 10 fractions (Total dose: 54 Gy).

Given the tumor’s high grade and potential for dissemination, adjuvant proton beam therapy of Pencil beam scanning was administered, consisting of craniospinal irradiation (CSI) at 36 Gy in 20 fractions (**Figure 2A**), followed by a local boost of 18 Gy in 10 fractions (Total dose: 54 Gy) (**Figure 2B**). This dose was selected based on established protocols for pediatric high-grade gliomas, balancing efficacy with the need to minimize long-term toxicity. No significant adverse events were observed during or immediately after treatment. Prior to proton beam therapy, T2-FLAIR imaging demonstrated patchy mild hyperintensity, and contrast-enhanced T1-weighted imaging (T1WI+C) showed heterogeneous mild-to-moderate enhancement of the lesion (**Figure 1B**). Following completion of proton beam therapy, the extent of contrast enhancement of the solid component was markedly reduced, while new perilesional brain edema was observed (**Figure 1C**).

Subsequent molecular profiling of the tumor tissue revealed a heterozygous BRCA2 mutation. Based on this finding, oral chemotherapy with the PARP inhibitor fluzoparib (50 mg, twice daily) was initiated as a bridging therapy. This off-label use was discussed extensively with the patient’s family, and written informed consent was obtained. The treatment was well-tolerated and was associated with a period of disease stability.

However, approximately 9 months post-proton beam therapy, the solid portion of the lesion had further decreased in size, although mild heterogeneous enhancement persisted (**Figure 1D**). To confirm the diagnosis and re-evaluate the lesion, a second craniotomy was performed, and histopathology again confirmed residual astroblastoma.

**Figure 3:**
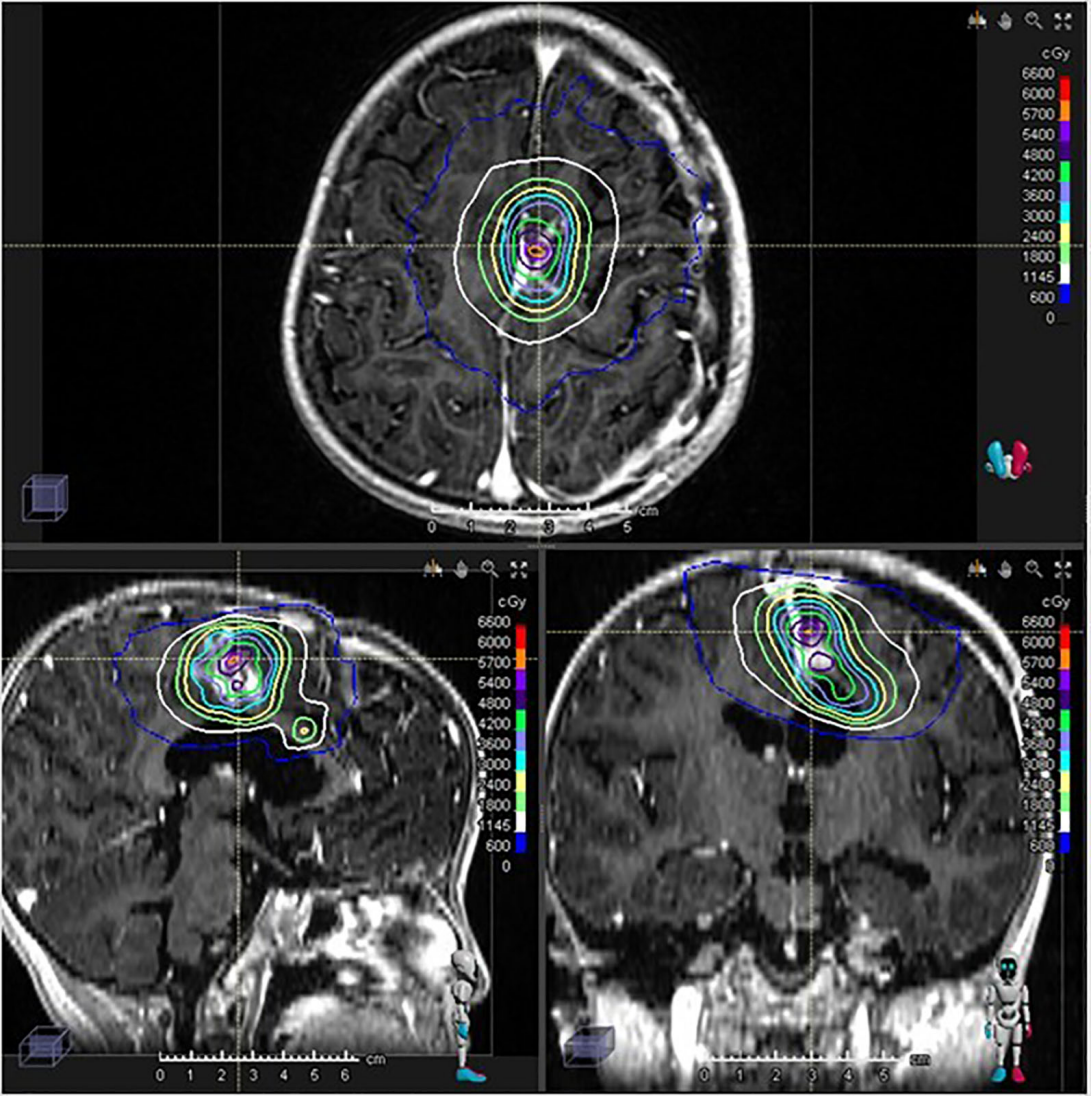
Gamma Knife stereotactic radiosurgery treatment plan and dose distribution. 30 Gy/5 fractions was performed.

The postoperative MRI showed near-total resection with small, nodular residual enhancing lesions. The interval between PBT and subsequent GKRS was dictated by this period of planned chemotherapy, observation, and the necessary second surgical intervention due to disease persistence. To address the residual disease, stereotactic radiosurgery (Gamma Knife) was performed. Two target volumes were identified within the enhancing lesion, and 30 Gy was delivered in 5 fractions to the 50% isodose line, using the frameless Gamma Knife ICON™ (Elekta) device (**Figure 3**).

## Diagnostic assessment

Diagnostic evaluation included neurological examination, computed tomography (CT), magnetic resonance imaging (MRI) (**Figure 1**), and histopathological analysis with immunohistochemistry following surgical resection. A key diagnostic challenge was differentiating post-radiation treatment effects (e.g., edema, non-specific enhancement) from true tumor progression on follow-up MRI scans after initial PBT, necessitating careful serial imaging and ultimately a second surgical confirmation. Postoperative pathological examination of the left frontal lobe lesion revealed a high-grade neuroepithelial tumor with both glial and ependymal differentiation, consistent with astroblastoma harboring MN1 alteration, classified as CNS WHO Grade 3. No other differential diagnoses were strongly considered after histopathology.

Immunohistochemistry profile:

- GFAP (partial positive)
- Olig2 (positive)
- S-100 (focally positive)
- Vimentin (positive)
- EMA (partially positive)
- D2-40 (positive)
- NeuN, CD34, EZHIP, TTF1-SPT24, CK, YAP-1, BCOR, P53, IDH1-R132H, H3K27M, EGFRvIII, L1CAM, C-myc, SOX10, Calretinin, Neurofilament (NF), and Synaptophysin were negative
- MAP2 (equivocal)
- Ki-67 labeling index: 25–30%
- ATRX (retained expression)
- H3K27me3 (retained expression)
- Cyclin D1 (positive)
- INI-1 and BRG-1 (retained)
- NTRK (1+), with a positive internal control
- Reticulin stain: negative

Molecular Analysis: Further molecular profiling identified a heterozygous BRCA2 mutation.

Prognosis: The tumor was classified as CNS WHO Grade 3, indicating a high-grade malignancy with associated prognostic implications.

## Follow-up and outcomes

By 18 months after subsequent Gamma Knife radiosurgery, the lesion no longer demonstrated a definite enhancing solid component; only linear enhancement at the margin of the cystic area was visible, with persistent mild FLAIR hyperintensity surrounding the lesion (**Figure 1E**). The mild perilesional FLAIR hyperintensity persisted but was asymptomatic and did not require any intervention, such as corticosteroids. No other significant radiation-related toxicities, such as radionecrosis, were observed during follow-up. The patient exhibited no neurological deficits and remained functionally independent in daily life activities. Patient’s guardian reported good quality of life with full participation in age-appropriate activities.

## Discussion

Although there are therapeutic strategies based on retrospective case series and individual case reports, the rarity of astroblastomas inherently restricts the feasibility of conducting extensive prospective clinical trials (15, 18). Therefore, the optimal treatment modality remains unclear. Maximum safe resection is the treatment of choice as like for other primary brain malignancies (6, 14). Radiation therapy is a crucial adjuvant treatment for high-grade astroblastoma, significantly improving survival rates (3, 4, 6, 8, 16, 18, 19). While its role in low-grade tumors is less clear, radiation can be used after subtotal resection to help control tumor growth (4, 18).

In their series 23 patients, Bonnin and Rubinstein reported that the sole patient who received radiotherapy following biopsy alone survived for 12 years post-diagnosis, indicating a potential benefit of radiotherapy in astroblastoma management (19). Furthermore, they observed that the only patient with high-grade astroblastoma who did not undergo postoperative radiotherapy had the shortest survival duration of 1.5 years, suggesting that the absence of adjuvant radiotherapy may be associated with poorer outcomes in high-grade cases.

Merfeld et al. conducted a retrospective analysis using data from the National Cancer Database to assess the impact of chemotherapy and radiotherapy on astroblastoma outcomes (18). The study found that among patients with high-grade astroblastoma, the 5-year OS of 33.3% for six patients who did not receive radiotherapy was significantly lower, compared to the 5-year OS of 84.6% for the 13 patients who did (p = 0.075), highlighting the potential survival benefit of adjuvant radiotherapy in high-grade astroblastoma cases.

Although recurrence in astroblastoma is most often local, dissemination to the spinal cord of astroblastomas have also been observed. Hirano H described a case of a 17-year-old male patient underwent surgery five times (four consecutive intracranial tumor removal surgeries and a final spinal tumor removal surgery) (20). After surgery for the spinal tumor, the patient underwent a course of ICE therapy. Three months after an additional 24 Gy local spinal irradiation was administered, his paraplegia improved slightly for a short period. Thereafter, follow-up head MRIs revealed new tumor recurrence in the basal ganglia. Cyber Knife radiotherapy was then selected to prevent neurological deficits by surgery. Stereotactic radiosurgery controlled the disease for several months, but the patient finally died as a result of the tumor in the middle of 2007.

In central nervous system tumors with a risk of dissemination, craniospinal irradiation (CSI) followed by local treatment, is a key treatment modality for many pediatric cancers, particularly brain tumors where radiotherapy is routinely delivered to the brain or entire craniospinal axis (21, 22). Pilocytic astrocytoma (PA), which is in the same group as astroblastoma, is reported to be disseminating throughout the central nervous system (23–25). In some case series and case report, craniospinal irradiation (CSI) was done for treatment of spinal drop metastasis with good outcome: overall survival time for 9 years, and the patients remained on follow-up (21, 23).

However, the risks of CSI in children include secondary cancers and cognitive impairments. Some studies showed that the different proportions of carcinogenic risks were observed in the lungs, breast, thyroid, stomach, liver, and other organs after different irradiation techniques or carcinogenic models for photon-based CSI radiotherapy (26–28). Bain et al. in 2013, introduced proton beam therapy for craniospinal irradiation (CSI) to minimize the exit dose, thereby concentrating radiation exposure exclusively on the craniospinal axis (23). This approach aimed to reduce radiation exposure to surrounding healthy tissues. Subsequent studies have demonstrated that proton beam CSI (p-CSI) can significantly decrease acute gastrointestinal and hematologic toxicities compared to conventional photon-based CSI, while maintaining comparable disease control outcomes (29).

A study conducted by Howell et al. compared the risks of radiogenic second cancers and cardiac mortality 18 pediatric medulloblastoma patients treated with passively scattered proton or field-in-field photon craniospinal irradiation (CSI), Proton CSI improved normal tissue sparing while also providing more homogeneous target coverage than photon CSI for patients across a wide age and BMI spectrum (30). Out of 24 evaluated parameters, (V5, V10, V15, and V20 in the esophagus, heart, liver, thyroid, kidneys, and lungs) Wilcoxon signed rank test results indicated 20 were significantly higher for photon CSI compared to proton CSI (p ≤ 0.05). Specifically, V15 and V20 in all six organs and V5, V10 in the esophagus, heart, liver, and thyroid were significantly higher with photon CSI.

In terms of dose distribution, Yoon et al. evaluated the dosimetric benefits for craniospinal irradiation in cancer in children using three-dimensional conformal radiotherapy (3D-CRT), tomotherapy (TOMO), and proton beam treatment (PBT) in the scattering mode (31). Compared with photon techniques, PBT showed improvements in most dosimetric parameters for CSI patients, with lower organ equivalent doses (OEDs) to organs at risk.

In this case, our management strategy was a sequential, multimodal approach tailored to the evolving clinical and molecular characteristics of the tumor. This included: 1) initial surgery, 2) adjuvant PBT, 3) molecularly-guided systemic therapy with a PARP inhibitor, 4) a second resection for persistent disease, and 5) high-dose salvage GKRS. The cornerstone of this strategy was the initial use of PBT. The physical properties of protons, specifically the Bragg peak, allowed for the delivery of a therapeutic dose to the craniospinal axis and tumor bed while significantly sparing surrounding healthy brain tissue.

After the first surgery, the postoperative MRI revealed a small residual enhancing lesion. Subsequently, considering the tumor’s propensity for dissemination, postoperative adjuvant radiotherapy with Proton CSI with a boost to the local tumor was chosen, to reduce the risk of damage to healthy tissue and create conditions for an increased biological dose to the tumor.

This tumor is highly malignant, with a tendency for residual disease, recurrence, and multiple relapses (6, 32). Therefore, in anticipation of secondary recurrence, a combination of repeat surgical resection and stereotactic radiotherapy (SRS) is considered. During the second course of radiotherapy, SRS is often employed for local control (17, 33), and in this context, the superior dose conformity of proton beam therapy—used during the initial treatment instead of conventional photon therapy—becomes a significant advantage, as it preserved a therapeutic window for safe and effective re-irradiation with high-dose GKRS for the residual disease—an option that might have been precluded by the higher integral dose of conventional photon therapy.

Gamma Knife stereotactic radiosurgery (GKRS) has advanced to provide high precision and expanded applicability for re-irradiation cases, including patients with a prior history of radiation therapy. A retrospective study conducted at the Mayo Clinic analyzed 174 patients with recurrent glioblastoma who underwent GKRS between 1991 and 2013. The median overall survival was 10.6 months following GKRS and 19.1 months from initial diagnosis, indicating that GKRS is a safe and modestly effective salvage treatment for recurrent glioblastoma (34).

In this case, Gamma Knife stereotactic radiosurgery (SRS) was administered to the local lesion, shortly after the second surgery of near-total resection. Head MRI 18 months after SRS demonstrated that the lesion remained within the irradiation field, with a decreased area of enhancement compared to pre-radiotherapy imaging, suggesting post-radiation changes. No signs of recurrence or metastasis were observed.

A novel aspect of this case was the use of a PARP inhibitor, fluzoparib, guided by the identification of a heterozygous BRCA2 mutation. While temozolomide is sometimes considered for high-grade gliomas, the presence of a known DNA damage repair pathway defect provided a strong rationale for a targeted approach. Although PARP inhibitor use in astroblastoma is not established, its selection here represents a personalized treatment strategy. This intervention was associated with a period of disease stability, bridging the patient to definitive local therapy.

Furthermore, definitive evidence regarding the optimal radiation dosage for astroblastoma is currently lacking. In the present case, initial treatment involved local irradiation delivering 54 Gy, a boost administered following CSI. Despite this regimen, the contrast-enhancing lesion persisted, and subsequent pathological examination after craniotomy 9 months later the proton beam therapy confirmed residual astroblastoma. This outcome suggests that a total dose of 54 Gy may have been inadequate for achieving local control in this specific instance. Considering that standard treatment protocols for high-grade gliomas typically employ doses around 60 Gy, this established practice could inform the determination of future prescribed doses for astroblastoma. Subsequently, Gamma Knife radiosurgery was administered at a dose of 30 Gy, a level consistent with dosages used for recurrent high-grade glioma irradiation. Notably, the patient has remained free from recurrence for 18 months following this intervention. Despite the limitation of being just one case report, with no large controlled studies yet available, it suggests a potential for controlling secondary recurrence in Astroblastoma.

Strengths and limitations: The strength of this report lies in detailing a multimodal approach combining advanced radiotherapy techniques (PBT and GKRS) for a rare, high-grade pediatric tumor with molecular confirmation (MN1-altered) and providing relatively long-term follow-up (18 months post-SRS). Limitations include its nature as a single case report, making generalizations difficult. The specific contribution of each treatment modality (PBT vs. chemo vs. GKRS) to the final outcome cannot be definitively isolated. Furthermore, optimal radiation doses and volumes for astroblastoma remain uncertain. Nonetheless, this case demonstrates the potential utility of a strategy that combines initial tissue-sparing PBT with molecularly targeted therapy and high-dose salvage SRS to manage this challenging disease.

Primary “take-away” lessons: This case highlights the challenge of managing high-grade pediatric astroblastoma and demonstrates the potential utility of a sequential multimodal strategy involving initial maximal safe resection followed by tissue-sparing adjuvant PBT. The conformal nature of PBT may be advantageous not only for reducing acute and long-term toxicity but also for preserving options for effective salvage therapy, such as high-dose SRS, in the event of residual or recurrent disease.

The patient’s perspective:

Our daughter’s diagnosis was overwhelming, but we felt supported by the medical team throughout the treatment process. The initial proton therapy seemed less harsh than we feared. Although needing further treatment with chemotherapy and Gamma Knife was difficult, we are incredibly grateful that she is doing well now, attending school, and living a normal life. We hope sharing her story can help other families facing this rare diagnosis.

## Conclusion

We presented a case of high-grade pediatric MN1-altered, BRCA2-mutated astroblastoma managed with maximal safe resection, adjuvant proton beam therapy including craniospinal irradiation, chemotherapy, and subsequent salvage Gamma Knife radiosurgery for residual disease. The clinical presentation, radiologic features, prognostic factors, and management strategies of astroblastomas were reviewed from the current literature. Given the rarity of astroblastoma, the optimal treatment strategy, particularly regarding radiation dose and modality, requires further investigation. We hope this case provides valuable insights into a potential multimodal approach for this challenging disease.

## Data Availability

The datasets presented in this study can be found in online repositories. The names of the repository/repositories and accession number(s) can be found in the article/supplementary material.
